# Subnatural-linewidth biphotons from a Doppler-broadened hot atomic vapour cell

**DOI:** 10.1038/ncomms12783

**Published:** 2016-09-23

**Authors:** Chi Shu, Peng Chen, Tsz Kiu Aaron Chow, Lingbang Zhu, Yanhong Xiao, M.M.T. Loy, Shengwang Du

**Affiliations:** 1 Department of Physics, The Hong Kong University of Science and Technology, Clear Water Bay, Kowloon, Hong Kong, China; 2 Department of Physics, State Key Laboratory of Surface Physics, Key Laboratory of Micro and Nano Photonic Structures, Fudan University, Shanghai 200433, China

## Abstract

Entangled photon pairs, termed as biphotons, have been the benchmark tool for experimental quantum optics. The quantum-network protocols based on photon–atom interfaces have stimulated a great demand for single photons with bandwidth comparable to or narrower than the atomic natural linewidth. In the past decade, laser-cooled atoms have often been used for producing such biphotons, but the apparatus is too large and complicated for engineering. Here we report the generation of subnatural-linewidth (<6 MHz) biphotons from a Doppler-broadened (530 MHz) hot atomic vapour cell. We use on-resonance spontaneous four-wave mixing in a hot paraffin-coated ^87^Rb vapour cell at 63 °C to produce biphotons with controllable bandwidth (1.9–3.2 MHz) and coherence time (47–94 ns). Our backward phase-matching scheme with spatially separated optical pumping is the key to suppress uncorrelated photons from resonance fluorescence. The result may lead towards miniature narrowband biphoton sources.

## Abstract (web summary)

Quantum-network protocols based on photon-atom interfaces have stimulated a great demand for single-photon sources with narrow bandwidth. Here the authors report the generation of entangled photon pairs with controllable bandwidth and coherence time from a Doppler-broadened hot atomic vapour cell.

## Graphical abstract

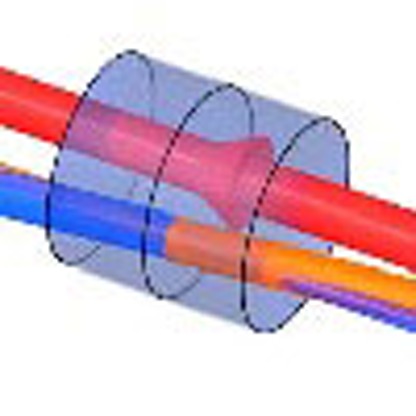

Biphotons (entangled photon pairs) are the benchmark tools in the field of quantum optics for probing fundamental quantum properties of light quanta such as the wave-particle duality and non-locality^1^. They have also played an important role in developing advanced technologies in quantum information processing^2^. The quantum-network protocols based on efficient photon–atom interaction require photons have bandwidth sufficiently narrower than the atomic natural linewidth^3^,^4^. Here we are interested in generating frequency-anticorrelated biphotons whose sum frequency is fixed and the biphoton bandwidth refers to the spectrum of individual photons. With a fast time-resolved detector, narrowband biphotons can be used to generate pure heralded single photons with the bandwidth equal to the biphoton bandwidth^5^. In earlier days, spontaneous parametric down conversion using nonlinear crystals^6^,^7^ and four-wave mixing in optical fibres^8^ were standard methods for producing biphotons. However, these biphotons have typically very wide bandwidth (>THz) and short coherence time (<ps), which make them extremely difficult for implementing photonic quantum information processing in an atomic-memory-based quantum network^9^,^10^. To solve this problem, many researches have focused on narrowing down the paired photon bandwidth by putting the nonlinear crystal inside an optical cavity^11^,^12^,^13^,^14^.

Subnatural-linewidth biphotons with controllable waveforms have been produced from spontaneous four-wave mixing (SFWM) in cold atoms (10–100 μK) assisted with electromagnetically induced transparency (EIT)^15^,^16^,^17^,^18^,^19^ or cavity^20^. However, cold-atom systems require expert knowledge in laser cooling and trapping. A cold-atom apparatus is not only expensive, but also large and complicated in its vacuum–optical–electronic–mechanical configuration. Moreover, operating cold atoms for producing paired photons requires a complex timing control with a low duty cycle^21^.

If a hot atomic vapour cell can be used as an alternative source to produce narrowband biphotons, the system size and operation can be markedly simplified and the cost will be significantly reduced. However, the use of hot atomic vapour cell for producing narrowband biphotons has not been as successful as those with cold atoms. In an early demonstration in 2005, Lukin *et al*.^22^ generated nonclassical correlated light pulses from a room temperature ^87^Rb atomic vapour cell with writing–reading pulse operation, but these photons are not time-frequency entangled and the photon number in each pulse is barely below the two-photon threshold. In this work, we focus on paired photon generation with time-frequency entanglement in continuous-wave operation mode. There have been some attempts in generating biphotons from hot atomic vapour cells, but with coherence time not exceeding 20 ns, corresponding to a bandwidth of >50 MHz that is much wider than the typical atomic natural linewidths^23^,^24^,^25^.

Here we demonstrate generating subnatural-linewidth biphotons using on-resonance SFWM in a hot ^87^Rb vapour cell assisted with EIT. Different from the off-resonance double-Raman scheme^26^ and diamond energy-level scheme^24^,^25^, where the photon bandwidth (∼500 MHz) is determined by the Doppler-broadening decoherence time (∼2 ns) of the excited atomic states, the EIT effect can significantly prolong the photon coherence time and narrow down the bandwidth^27^. However, when directly applying the EIT-assisted SFWM scheme to a hot vapour cell, there is a serious noise problem: uncorrelated photons generated from resonance Raman scattering of the strong coupling laser field overwhelm the entangled photon pairs. To overcome this problem, we coat the inner wall of the cell with paraffin to increase the atomic ground-state coherence time and apply an additional strong optical-pumping beam to suppress the on-resonance scattering of the coupling field. The optical-pumping beam is spatially separated from the SFWM volume and does not interfere with the biphoton generation. This noise reduction together with other optical filtering allows us observing biphotons with a high contrast ratio.

## Results

### Biphoton generation with optical pumping

**Figure 1:**
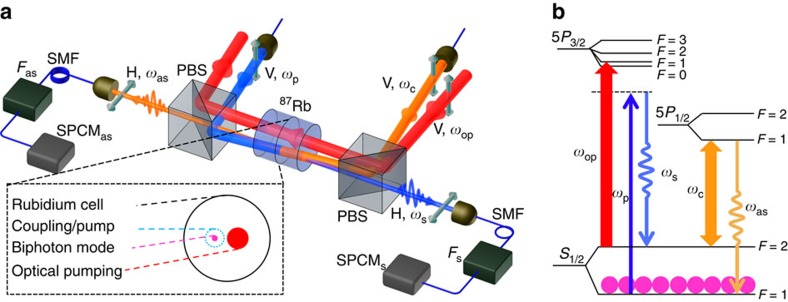
Generating narrowband biphotons from a hot ^87^Rb vapour cell. (**a**) Experimental set-up. In the presence of counter-propagating vertically (V) polarized pump (780 nm, *ω*_p_) and coupling (795 nm, *ω*_c_) laser beams, horizontally polarized (H) Stokes (780 nm, *ω*_s_) and anti-Stokes (795 nm, *ω*_as_) photon pairs are spontaneously generated and coupled into two opposing single-mode fibres (SMF). Then, they pass through optical frequency filters (*F*_s_ and *F*_as_), and are detected by two single-photon counting modules (SPCM_s_ and SPCM_as_). The coincidence counts are recorded by a time-to-digit converter (Fast Comtec P7888). Two polarizing beam splitters (PBSs) are used as polarization filters to distinguish the paired photons from the two driving laser beams. The fibre–fibre coupling efficiency and SPCM detection efficiency are 80% and 50%, respectively. Each optical frequency filter composes of a wide-band line filter and a narrowband etalon Fabry–Perot cavity filter. The etalon filters have free spectrum range (FSR)=13.6 GHz. The bandwidth, transmission efficiency and the extinction ratio of the frequency filters are 350 MHz, 80% and 60 dB for *F*_s_, and 80 MHz, 30% and 40 dB for *F*_as_, respectively. In the inset, we make a zoom-in on the transverse cross-section of cell to clearly show the beam profiles of all incident lasers and the biphoton mode. The biphoton mode has a waist diameter (1/*e*^2^) of 250 μm focused in the middle of the 0.5-inch-long cell. The collimated coupling and pump laser beams are counter propagating and have the same 1/*e*^2^ beam diameter of 1.4 mm. The optical-pumping beam has a 1/*e*^2^ diameter of 2 mm and does not overlap with the SFWM volume enclosed by the pump and coupling beams. The cell inner diameter is 10 mm. (**b**) The relevant ^87^Rb atomic energy-level diagram for backward SFWM and optical pumping. The strong optical-pumping laser is used to optically pump the atoms from the level |5*S*_1/2_, *F*=2〉 to |5*S*_1/2_, *F*=1〉, to suppress the on-resonance Raman scattering of the coupling beam.

We produce subnatural-linewidth biphotons from a paraffin-coated ^87^Rb vapour cell at 63 °C, as illustrated in Fig. 1. The details of the experimental set-up are described in the Methods section. In presence of two counter-propagating pump (*ω*_p_) and coupling (*ω*_c_) laser beams, backward and phase-matched Stokes (*ω*_s_) and anti-Stokes (*ω*_as_) photon pairs are spontaneously generated. After spatial and frequency filtering, these photons are detected by two single-photon counting modules (SPCM_s_ and SPCM_as_). We find the major noise source of uncorrelated photons is the on-resonance Raman scattering of the coupling field following the transition |5*S*_1/2_, *F*=2〉→|5*P*_1/2_, *F*=1〉→|5*S*_1/2_, *F*=1〉. These photons have the same central frequency and polarization as the anti-Stokes photons, and cannot be filtered away by the polarization and frequency filters. To clean up the residual atoms in the level |5*S*_1/2_, *F*=2〉, we apply a strong optical-pumping beam (*ω*_op_) on the transition |5*S*_1/2_, *F*=2〉→|5*P*_3/2_, *F*=1〉. In order not to interfere with the SFWM transitions, the optical-pumping beam is aligned parallel to the Stokes–anti-Stokes mode without spatial overlap. The atoms in the level |5*S*_1/2_, *F*=2〉 are optically pumped to the ground level |5*S*_1/2_, *F*=1〉, and thus the Raman scattering on the anti-Stokes channel is suppressed, owing to the long ground-state coherence time because of the paraffin coating.

**Figure 2:**
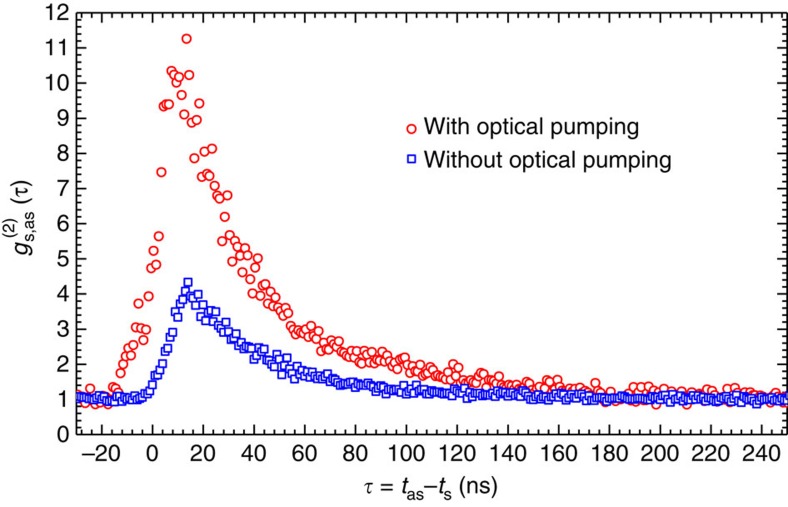
Biphoton cross-correlation function with and without optical pumping. The control experiment is performed under 6 mW pump laser power and 27 mW coupling laser power. The red circles are measured in presence of the spatially separated optical-pumping beam (32 mW) and the blue squares are measured without the optical-pumping beam.

To confirm that the spatially separated optical-pumping beam reduces the on-resonance Raman scattering of the coupling laser beam, we perform a control experiment of biphoton generation with and without optical pumping. The powers of the pump and coupling laser beams are 6 and 27 mW, respectively. We observe that, after switching on the optical-pumping beam (32 mW), the photon detection rate on the anti-Stokes channel drops from 12,000 s^−1^ to 3,600 s^−1^, while the photon-pair rate is nearly unaffected. The contrast ratio between the biphoton coincidence (signal) and the accidental coincidence (noise) can be characterized by the normalized two-photon correlation functions 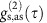, which are plotted in Fig. 2. It clearly shows that the peak value 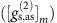 of the normalized two-photon correlation or the biphoton–noise contrast ratio increases by a factor of ∼3 when we switch on the optical-pumping beam due to the reduction of the accidental coincidence counts.

### Subnatural-linewidth biphotons

**Figure 3:**
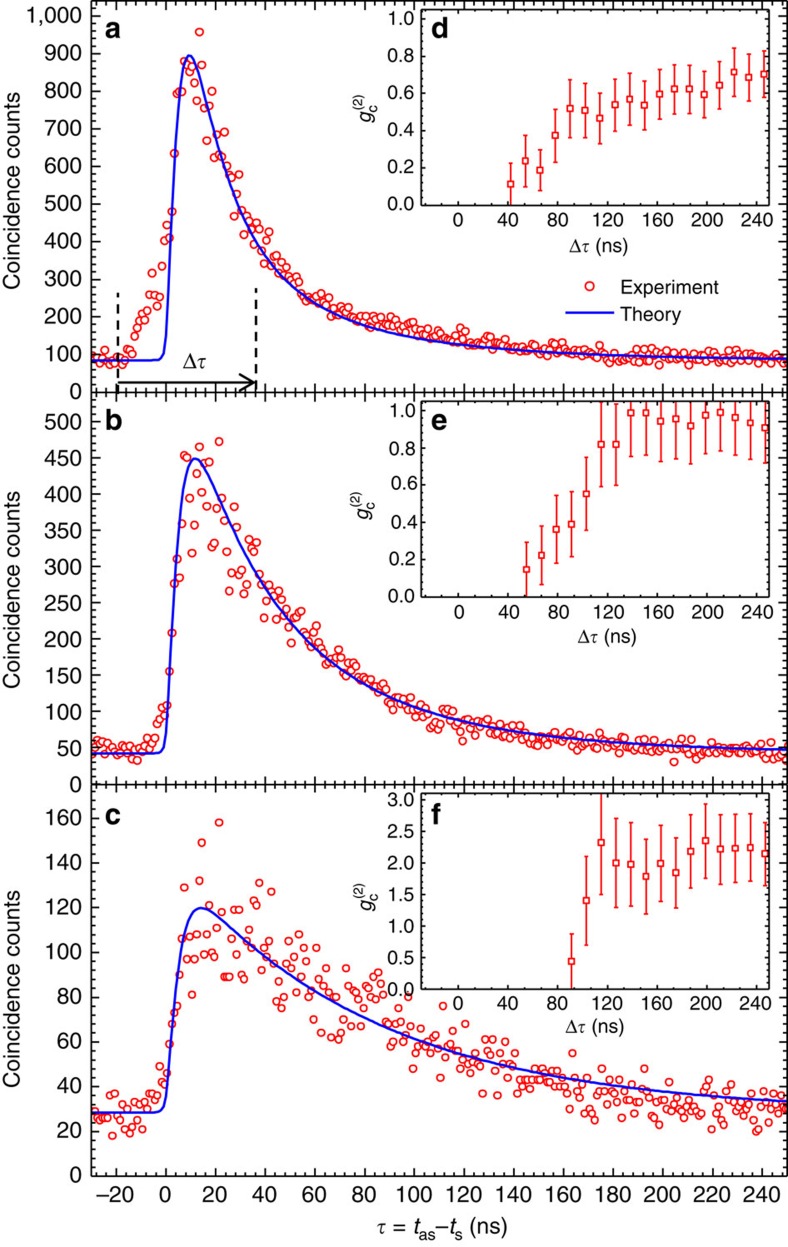
Biphoton waveforms with controllable correlation time. (**a**–**c**) Two-photon coincidence counts, collected over 600 s with 1 ns bin width, as a function of the relative time delay 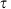 between paired Stokes and anti-Stokes photons. The incident pumping laser power is fixed at 6 mW, while the incident coupling laser powers of **a**,**b** and **c** are 27, 9 and 1 mW, respectively. The red circles are the experimental data. The solid blue curves are obtained numerically with the experimental parameters by taking into account the Doppler effect (Supplementary Note 1). The corresponding measured conditional autocorrelation function 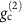 of heralded single anti-Stokes photons are plotted in **d**–**f**. The error bars are s.d.'s resulting from the statistical uncertainties of coincidence counts that can be reduced with longer data taking time.

Figure 3 shows the biphoton waveforms. We fix the pump laser power at 6 mW and vary the coupling laser power, which is 27, 9 and 1 mW for Fig. 3a–c, respectively. As expected, the two-photon correlation time becomes longer as we reduce the coupling laser power for narrower EIT window. Shown in Fig. 3a–c, the biphoton waveforms are exponential decay. The 1/*e* correlation times are 47, 60 and 94 ns, for Fig. 3a–c, respectively, all exceeding the natural lifetime 26.5 ns of Rb 5*P* excited states. The blue theoretical curves are obtained numerically by taking into account the Doppler effect (Supplementary Note 1) and agree well with the experiment. This agreement allows us to extract the biphoton temporal wave function and joint spectrum. The bandwidths of these biphotons are 3.2, 2.6 and 1.9 MHz, for Fig. 3a–c, respectively (Supplementary Fig. 1). They are substantially narrower than the natural linewidth of 6 MHz of Rb D1/D2 lines.

To characterize the nonclassical property of the photon-pair source, we confirm its violation of the Cauchy–Schwarz inequality^28^. Normalizing the coincidence counts to the accidental background floor in Fig. 3a–c, we get the normalized cross-correlation function 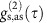 with maximum values 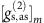
=11±1, 11±2 and 6±1. With the measured autocorrelations 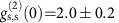 and 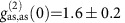, we obtain violation of the Cauchy–Schwarz inequality 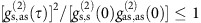 by factors of 38±8, 38±11 and 11±3, for Fig. 3a–c, respectively. We further verify the quantum nature of heralded anti-Stokes photons by measuring their conditional autocorrelation function 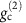 (ref. 29). An ideal single-photon source gives 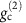
=0. A two-photon Fock state gives 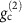
=0.5, and a coherent state gives 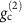
=1. The measured 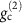 as a function of coincidence window width 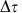 are plotted as Fig. 3d–f, which are below the two-photon threshold within their coherence time.

**Figure 4:**
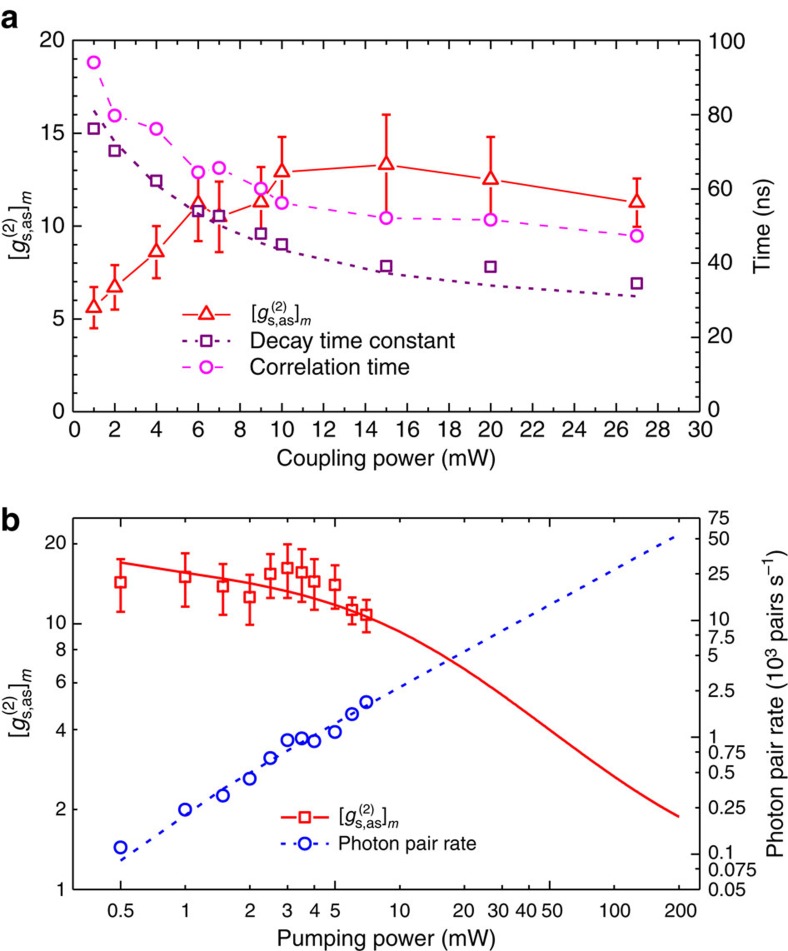
Biphoton generation with controllable properties. (**a**) The biphoton properties versus the coupling laser power. The pump laser power is fixed at 6 mW. The red triangular data represent the maximum of the normalized cross-correlation function, 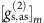. The decay time constant (purple, square) and 1/*e* correlation time (magenta, circular) are also plotted. The 1/*e* correlation time takes into account the rise time of the biphoton waveform in its rising edge. (**b**) 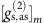 and biphoton generation rate versus pump laser power. The fibre coupling efficiencies, filter transmissions and SPCM quantum efficiencies have been all taken into account to obtain the photon-pair generation rate. The coupling laser power is fixed at 27 mW. The solid curve is a theoretical plot obtained by taking into account the uncorrelated noise photons (Supplementary Note 2). The error bars represent the s.d.'s resulting from statistical uncertainties of measured coincidence counts.

As we reduce the coupling laser power, the EIT bandwidth becomes narrower and the dispersion induced phase mismatching further constrains the biphoton joint spectrum^27^. Therefore, the biphoton bandwidth and coherence time are not determined by the lifetime of the excited states even though the photon pairs are indeed generated spontaneously. Figure 4a shows the measured decay time constant (square) and 1/*e* correlation time (circle) of the biphoton waveform as functions of coupling laser power. The longest correlation time at 1 mW coupling power approaches ∼100 ns. On the other side, 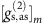, the maximum value of the normalized cross-correlation function, decreases as we reduce the coupling power. Figure 4b shows the 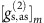 and photon-pair generation rate as functions of the pump power. While the photon-pair rate is proportional to the pump laser power, the 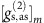 drops at a high pump power. Limited by our available pump laser power of 7 mW, we produced ∼2,000 pairs per second. As the threshold of the 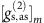 is 2.0 for violating the Cauchy–Schwarz inequality, the nonclassical property of the photon source is still preserved at 170 mW pump power, which corresponds to a generation rate of∼47,000 pairs per second.

## Discussion

In summary, we demonstrate generation of subnatural-linewidth biphotons from a hot paraffin-coated ^87^Rb vapour cell using EIT-assisted SFWM. The biphoton coherence time, controlled by the coupling laser power, can be as long as 94 ns. The corresponding bandwidth of 1.9 MHz is substantially narrower than the natural linewidth 6 MHz of Rb D1/D2 transitions. It can be used to generate nearly pure heralded single photons^5^. The exponential waveform with tunable time constant is perfect for interacting with atoms^30^ and coupling to an optical cavity^31^. In this work, the heralding efficiency of the photon pairs is 3.1%. This is determined by the small optical depth of ∼1 of the atomic vapour cell, which is limited by the maximally allowed temperature (<70 °C) of the paraffin coating. If we can further increase the cell temperature while maintaining a low spin relaxation rate, we expect to improve the heralding efficiency and generate biphotons with richer waveforms by engineering the spatial profile of the pump beam, as those demonstrated in cold atoms^19^.

The two key elements to make the SFWM narrowband biphoton generation feasible are the paraffin coating and the spatially separated optical pumping. The long ground-state coherence time preserved by the paraffin coating enables the efficient optical pumping, which is spatially separated from the biphoton generation volume, for the flying atoms without interfering the SFWM transitions. As a general state preparation method, the technology demonstrated here can be immediately applied to reduce incoherent photon noise thus improve the fidelity of the Raman-based quantum memory^32^. Future improvements could include improving the quality of optical frequency filtering (etalon Fabry–Perot cavity, polarization filter, spatial-mode filter) and optimizing the power and spatial profile of the optical pump beam.

As compared with the cold-atom experiments^33^,^34^, the hot atomic vapour cell configuration is much simpler for operation and maintenance, and it is a continuous biphoton source. Our demonstration may lead to miniature narrowband biphoton sources based on atomic vapour cells for practical quantum applications and engineering.

## Methods

### Experimental set-up

The experimental set-up and associated atomic energy-level diagram are illustrated in Fig. 1. A paraffin-coated ^87^Rb (99% enrichment purity, Precision Glassblowing Inc) vapour cell is placed in a temperature-stabilized hot-air heating oven, which is not shown in Fig. 1a, and is set at 63 °C with fluctuation <0.2 °C. The length of the vapour cell is *L*=0.5 inch and its inner diameter is *d*=10 mm. The longitudinal orientation of the cell is from east to west and there is no magnetic shielding in this experiment. The SFWM process is driven by two laser fields: the pump laser (D2 line: 780 nm, *ω*_p_) is locked to the ^85^Rb transition |5*S*_1/2_, *F*=2〉→|5*P*_3/2_, *F*=3〉, which is red detuned by 2.7 GHz from the ^87^Rb transition |5*S*_1/2_, *F*=1〉→|5*P*_3/2_, *F*=2〉, and the coupling laser (D1 line: 795 nm, *ω*_c_) is on resonance to the transition |5*S*_1/2_, *F*=2〉→|5*P*_1/2_, *F*=1〉. The vertically polarized pump and coupling laser beams are counter propagating with the same 1/*e*^2^ beam diameter of 1.4 mm. Backward, horizontally polarized, Stokes (780 nm, *ω*_s_) and anti-Stokes (795 nm, *ω*_as_) photon pairs are spontaneously generated, coupled into two opposing single-mode fibres, passing through optical frequency filters (*F*_s_ and *F*_as_), and detected by two single-photon counting modules (SPCM_s_ and SPCM_as_, Excelitas/PerkinElmer SPCM-AQRH-16-FC). The two-photon coincidence counts are recorded by a time-to-digit converter (Fast Comtec P7888) with a temporal bin width of 1 ns. Two polarization beam splitters are used as polarization filters to distinguish the paired photons from the two driving laser beams. The spatially separated optical pumping is implemented by applying a strong vertically polarized optical-pumping beam (*ω*_op_) that is on resonance to the transition |5*S*_1/2_, *F*=2〉→|5*P*_3/2_, *F*=1〉. The optical-pumping beam is aligned parallel to the pump-coupling beams without overlap. The laser beam profiles on the cross-section of the cell are shown in the inset of Fig. 1a. The optical-pumping beam, with a power of 32 mW, has a 1/*e*^2^ beam diameter of 2 mm. The Stokes and anti-Stokes single-mode diameter on the cell centre is 250 μm. To further separate the generated photon pairs from the two driving laser beams, the pump and coupling laser beams are aligned with an angle of ∼0.5° to the Stokes and anti-Stokes directions.

### Normalized cross- and autocorrelation functions

The normalized two-photon cross-correlation function is defined as 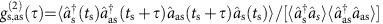, where 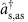 and 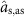 are the creation and annihilation operators of the Stokes and anti-Stokes fields, respectively. The experimental 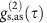 is obtained by normalizing the two-photon coincidence counts to the flat background floor of accidental coincidence counts. The normalized autocorrelation functions are defined as 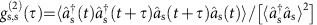 and 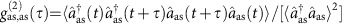. The autocorrelation functions are measured using a fibre beam splitter.

### Theoretical calculation of biphoton waveforms

The theoretical curves in Fig. 3 are obtained numerically following the Schrodinger picture approach^27^ by integrating over the Doppler-broadening profile (Supplementary Note 1). The solid theoretical curve in Fig. 4b is obtained by taking into account the uncorrelated noise photons (Supplementary Note 2).

### Data availability

The data that support the findings of this study are available from the corresponding author on request.

## Additional information

**How to cite this article:** Shu, C. *et al.* Subnatural-linewidth biphotons from a Doppler-broadened hot atomic vapour cell. *Nat. Commun.* 7:12783 doi: 10.1038/ncomms12783 (2016).

## Supplementary Material

Supplementary Information Supplementary Figure 1, Supplementary Notes 1-2 and Supplementary References
