# Supplementary material for: Subnatural-linewidth biphotons from a Doppler-broadened hot atomic vapour cell
Source: Nat Commun. 2016 Sep 23;7:12783. doi: 10.1038/ncomms12783 (PMC5036144; doi:10.1038/ncomms12783)
Supplement: Supplementary Information — Supplementary Figure 1, Supplementary Notes 1-2 and Supplementary References [file ncomms12783-s1.pdf]

Supplementary Figure 1

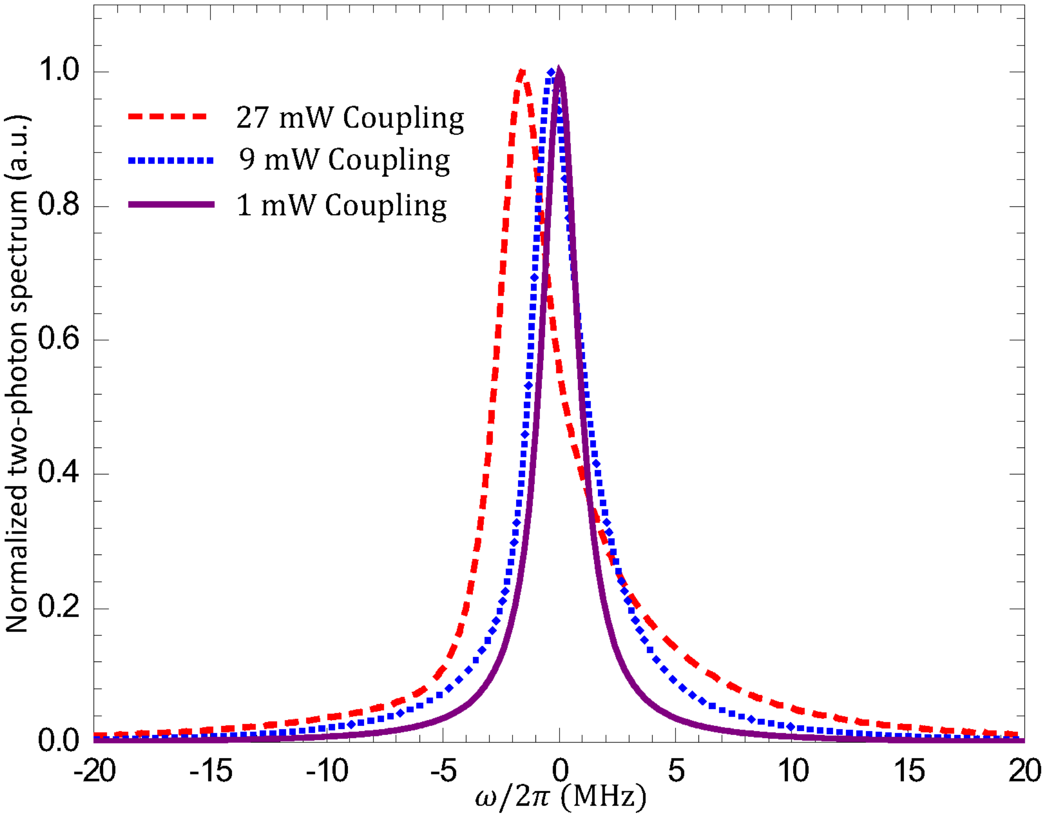

Normalized biphoton power spectrum. The red dash line, the blue dot line and the purple solid line represent the cases with 27 mW, 9 mW and 1 mW coupling laser power, respectively. The pump power is fixed at 6 mW.

Supplementary Note 1

Theory of biphoton generation from a Doppler-broadened Atomic Medium

In this note we provide the theory model in calculating the theoretical curves in Fig. 3 of the main manuscript. The relevant ^{87}Rb spontaneous four-wave mixing (SFWM) energy level diagram is shown in Fig. 1 of the main text. The nonlinear atomic medium is driven by two classical laser fields: ω_p, the pump beam laser field, and ω_c, the coupling beam laser field. In the theoretical derivation, we take the following notations: $|1\rangle = |5S_{1/2}, F=1\rangle$, $|2\rangle = |5S_{1/2}, F=2\rangle$, $|3\rangle = |5P_{1/2}, F=1\rangle$, and $|4\rangle = |5P_{1/2}, F=2\rangle$. Because the pump laser is far detuned by more than 2 GHz from the D2 transitions $|1\rangle \rightarrow |5P_{3/2}, F=0,1,2\rangle$ as compared to their natural linewidth and Doppler broadening, these hyperfine levels are treated as one effective state $|5\rangle = |5P_{3/2}, F=0,1,2\rangle$ for theoretical simplicity. The coupling laser is on resonance to the D1 transition $|2\rangle \rightarrow |3\rangle$. The paired Stokes ($\omega_s$) and anti-Stokes ($\omega_{as}$) photons are spontaneously generated.

Following the spontaneous four-wave mixing (SFWM) formalism[1], we have the following expression for the biphoton temporal wavefunction

$$\Psi(t_s, t_{as}) = \psi(\tau) e^{-i(\varpi_s t_s + \varpi_{as} t_{as})}, \tag{1}$$

where ϖ_s and ϖ_{as} are the central angular frequencies of the Stokes and anti-Stokes photons. The relative wavefunction is

$$\psi(\tau = t_{as} - t_s) = \frac{L}{2\pi} \int \mathrm{d}\omega \kappa(\omega) \Phi(\omega) e^{-i\omega\tau}, \tag{2}$$

where

$$\omega = \omega_{as} - \varpi_{as} = -(\omega_s - \varpi_s), \tag{3}$$

and the nonlinear coupling coefficient $\kappa(\omega)$ and the longitudinal detuning function $\Phi(\omega)$ are

$$\kappa(\omega) = \frac{\sqrt{\varpi_{as}\varpi_s}}{2ic_0} \chi^{(3)}(\omega) E_p E_c, \tag{4}$$

$$\Phi(\omega) = \mathrm{sinc}\left(\frac{\Delta k L}{2}\right) e^{i(k_{as}+k_s)L/2}, \tag{5}$$

where k is the wave number and$\Delta k = (k_{as} - k_s) - (k_c - k_p)$ is the wave number mismatching in the medium.

Because the pump-coupling beams and Stokes-anti-Stokes modes are aligned nearly collinear with a small angle of 0.5^{o}, here we count only one-dimensional Doppler effect. For the coupling (ω_c) and anti-Stokes (ω_{as}) transitions, we take into account both hyperfine levels of $5P_{1/2}$: $|3\rangle = |5P_{1/2}, F=1\rangle$, and $|4\rangle = |5P_{1/2}, F=2\rangle$. The wave numbers of Stokes and anti-Stokes, linear and third-order nonlinear susceptibilities are

$$k_{\mathrm{s}}(\omega) = (\varpi_{\mathrm{s}}/c)\sqrt{1+\chi_{\mathrm{s}}(\omega)}, \tag{6}$$

$$k_{\mathrm{as}}(\omega) = (\varpi_{\mathrm{as}}/c)\sqrt{1+\chi_{\mathrm{as}}(\omega)} \tag{7}$$

$$\chi_{\mathrm{s}}(\omega) = \int f(v)\mathrm{d}v \frac{N|\mu_{25}|^2}{\epsilon_0\hbar}\frac{|\Omega_{\mathrm{p}}|^2}{\Delta_5(\omega,v)^2+\gamma_5^2}$$

$$\{\frac{[\Delta_3(\omega,v)-i\gamma_3]}{|\Omega_{\mathrm{c3}}|^2-4[\Delta_2(\omega,v)-i\gamma_2][\Delta_3(\omega,v)-i\gamma_3]}+\frac{[\Delta_4(\omega,v)-i\gamma_4]}{|\Omega_{\mathrm{c4}}|^2-4[\Delta_2(\omega,v)-i\gamma_2][\Delta_4(\omega,v)-i\gamma_4]}\}, \tag{8}$$

$$\chi_{\mathrm{as}}(\omega) = \int f(v)\mathrm{d}v \frac{4N}{\epsilon_0\hbar}[\Delta_2(\omega,v)+i\gamma_2]$$

$$\{\frac{|\mu_{13}|^2}{|\Omega_{\mathrm{c3}}|^2-4[\Delta_2(\omega,v)+i\gamma_2][\Delta_3(\omega,v)+i\gamma_3]}+\frac{|\mu_{14}|^2}{|\Omega_{\mathrm{c4}}|^2-4[\Delta_2(\omega,v)+i\gamma_2][\Delta_4(\omega,v)+i\gamma_4]}\}, \tag{9}$$

$$\chi^{(3)}(\omega) = \int f(v)\mathrm{d}v \frac{N\mu_{25}\mu_{15}}{\epsilon_0\hbar^3}\frac{1}{\Delta_5(\omega,v)+i\gamma_5}$$

$$\{\frac{\mu_{13}\mu_{23}}{|\Omega_{\mathrm{c3}}|^2-4[\Delta_2(\omega,v)+i\gamma_2][\Delta_3(\omega,v)+i\gamma_3]}+\frac{\mu_{14}\mu_{24}}{|\Omega_{\mathrm{c4}}|^2-4[\Delta_2(\omega,v)+i\gamma_2][\Delta_4(\omega,v)+i\gamma_4]}\}, \tag{10}$$

where 1D Maxwell-Boltzmann distribution is

$$f(v) = \sqrt{\frac{m_{\mathrm{Rb}}}{2\pi k_{\mathrm{B}}T}}\exp\,[-\frac{m_{\mathrm{Rb}}v^2}{2k_{\mathrm{B}}T}]. \tag{11}$$

Here k_{B} is the Boltzmann constant and T is the cell temperature. The atomic density is estimated from its saturated vapor pressure[2] at the corresponding temperature T:

$$N = P/(k_{\mathrm{B}}T). \tag{12}$$

μ_{ij} is the dipole matrix element between $|i\rangle$ and $|j\rangle$, γ_2 is experimental fitted parameter considering dephasing and relaxation between two hyperfine ground states $|1\rangle$ and $|2\rangle$, $\gamma_{\mathrm{i=3,4,5}}$ is decay rate of excited states $|i\rangle$; and Rabi frequency of coupling and pump are Ω_{ci} (i = 3,4 for different configuration) and Ω_{p} respectively. Detunings of the light fields and corresponding energy levels are

$$\Delta_2(\omega,v) = (\varpi_{\mathrm{as}}-\varpi_{\mathrm{c}})(1-v/c)-\omega_{21}+\omega(1-v/c), \tag{13}$$

$$\Delta_3(\omega,v) = \varpi_{\mathrm{as}}(1-v/c)-\omega_{31}+\omega(1-v/c), \tag{14}$$

$$\Delta_4(\omega,v) = \varpi_{\mathrm{as}}(1-v/c)-\omega_{41}+\omega(1-v/c), \tag{15}$$

$$\Delta_5(\omega,v) = \varpi_{\mathrm{p}}(1+v/c)-\omega_{51}. \tag{16}$$

The biphoton coincidence counts can be calculated from

$$C(\tau) = \beta|\psi(\tau)|^2\Delta t_{\mathrm{bin}}Time, \tag{17}$$

where β is the two-photon joint detection efficiency, Δt_{bin} is the time-bin width, and $Time$ is the collection time. As shown in Fig.3 of the main manuscript, the theory agrees well with the experiment in the biphoton waveform. This agreement allows us to extract the biphoton spectrum $|\kappa(\omega)\Phi(\omega)|^2$. The normalized biphoton power spectrums for the cases in Figs. 3a-3c of the main manuscript are shown in Supplementary Fig. 1. The FWHM bandwidths are 3.2 MHz, 2.6 MHz and 1.9 MHz for 27 mW, 9 mW and 1 mW coupling laser powers, respectively.

Supplementary Note 2

$\left[g_{\mathrm{s,as}}^{(2)}\right]_{\mathrm{m}}$ vs pump power

In this Supplementary Note, we give the formalism for the obtaining the solid curve in Fig.4b of the main text.

As the pump laser is far detuned from the transition$|1\rangle \rightarrow |5\rangle$, the photon pair generation rate is proportional to the pump laser power:$R = \eta P$, where R is the pair photon generation rate and P is the pump laser power. This is confirmed by the linear dependence of photon pair rate as function of pump power in Fig.4b of the main manuscript. The major uncorrelated photons, as we measured, are still on the anti-Stokes channel with a rate of $R_{asn} = \xi P + R_{as0}$, including ξP the uncorrelated photons resulted from the depopulation caused by the pump laser and R_{as0} the resonance Raman scattering at zero pump power. The uncorrelated photon rate $R_{sn} = \zeta P$ on the Stokes channel is also promotional to the pump power. Then the maximum value of cross correlation function $\left[g_{\mathrm{s,as}}^{(2)}\right]_{\mathrm{m}}$ with an exponential-decay shape can be calculated as

$$\left[g_{\mathrm{s,as}}^{(2)}\right]_{\mathrm{m}} = 1 + \frac{R/\tau_{\mathrm{b}}}{(R+R_{\mathrm{sn}})(R+R_{\mathrm{asn}})} = 1 + \frac{\eta}{(\eta+\zeta)[(\eta+\xi)P+R_{\mathrm{as0}}]\tau_{\mathrm{b}}}, \tag{18}$$

where τ_{b} is the decay time constant of the biphoton waveform. The above equation is used to plot the solid curve in Fig.4b with the following experimentally determined parameters: $\eta = 266$ s ^{1}mW ^{1}, $\zeta = 9800$ s^{-1}mW^{-1} , ξ =3330s^{-1} mW^{-1}, $R_{\mathrm{as0}} = 44470$ s^{-1}, and $\tau_{\mathrm{b}} = 34$ ns.

Supplementary References

1 Du, S., Wen, J. & Rubin, M. H. Narrowband biphoton genration near atomic resonance. *J. Opt. Soc. Am. B* **25**, C98-C108 (2008).

2 Steck, D. A. Rubidium 87 D Line Data, available online at http://steck.us/alkalidata (revision 2.0.1, 2008)
